# Improving word embeddings in Portuguese: increasing accuracy while reducing the size of the corpus

**DOI:** 10.7717/peerj-cs.964

**Published:** 2022-07-18

**Authors:** José Pedro Pinto, Paula Viana, Inês Teixeira, Maria Andrade

**Affiliations:** 1 INESC TEC, Porto, Portugal; 2 School of Engineering, Polytechnic of Porto, Porto, Portugal; 3 Faculty of Engineering, University of Porto, Porto, Portugal

**Keywords:** Natural language processing, Machine learning, Multimedia systems, Context awareness, Word2Vec

## Abstract

The subjectiveness of multimedia content description has a strong negative impact on tag-based information retrieval. In our work, we propose enhancing available descriptions by adding semantically related tags. To cope with this objective, we use a word embedding technique based on the Word2Vec neural network parameterized and trained using a new dataset built from online newspapers. A large number of news stories was scraped and pre-processed to build a new dataset. Our target language is Portuguese, one of the most spoken languages worldwide. The results achieved significantly outperform similar existing solutions developed in the scope of different languages, including Portuguese. Contributions include also an online application and API available for external use. Although the presented work has been designed to enhance multimedia content annotation, it can be used in several other application areas.

## Introduction

In the last couple of decades, a tremendous progress in information and communications technology has been witnessed. Nowadays, anyone can become a multimedia content producer and easily disseminate content through the Internet. As a result, the amount and diversity of media content available to any consumer have increased at an exponential rate. While this has clear advantages, it also raises several challenges: the need to conveniently annotate content to be valuable and usable. Ideally, keywords and annotations should be generated consistently so that only the content relevant to the user is included in the results’ list. The completeness of this list should be ensured so that all similar or related content is included. To enable increasing such consistency, researchers worldwide have struggled to deliver common and open standards for the representation of multimedia metadata. However, irrespective of such efforts, the truth is that in the real world the use of keywords continues to be quite heterogeneous and very much subjective, depending on the person who manually annotated content or who develops the software to extract keywords automatically. Consequently, when performing a keyword-based search, many relevant contents will not be included in the list of results returned to the user. Our work departs from this well-known problem and proposes a solution based on the assumption that different tags used by different annotation processes on similar/related contents will share some hypernyms or synonyms. Hence, by predicting the contextualized co-occurrence of tags assigned to online content, it will be possible to find similar or related content and thus to build more meaningful/complete results’ lists for keyword-based searches. In previous work, we have exploited the use of semantic dictionaries that enable extracting semantically related concepts (Viana & Pinto, 2017) to enhance public-contributed metadata (Pinto & Viana, 2013; Pinto & Viana, 2015). Additionally, a methodology to improve YouTube content descriptions, by using this metadata, was proposed (Pinto & Viana, 2018). The work presented in this paper exploits Natural Language Processing (NLP) and neural networks to improve existing solutions in annotation tasks, but it can also be used for other purposes, which will be described in more detail in this document.

Different approaches can be adopted to determine vector representations of words and to measure their quality. Word embeddings is an approach that has received much attention lately because of its ability to represent similar words as nearby points in a vector space. Each word is represented by a vector containing a number of features extracted from the word and its context, considered as a part of a text corpus (Joulin et al., 2016; Turian, Ratinov & Bengio, 2010). A word embedding is then a contextualized vector representation of a word. By using vectors to represent words, the similarity between pairs of words can be determined by computing, for example, the cosine distance between the two vectors. Such vectorised representations can thus provide efficient generalizations when the objective is to compare lexical items.

A common approach to obtain accurate and consistent word embeddings is to use neural networks that receive as input a text corpus. The larger the input text corpus, the better the quality of the model and thus of the generated word embeddings. Such models are usually named as distributional semantic models (Lenci, 2018) based on the assumption that the statistical distribution of linguistic items in context (*i.e.*, within a text corpus) is highly correlated with their semantic value. In practice, this translates into the hypothesis that semantically similar words are likely to be found in similar contexts with similar probabilities. Neural networks that have been used for this purpose include Word2Vec, FastText or Glove (Joulin et al., 2016; Mikolov et al., 2013; Inc., 2020; Bojanowski et al., 2017; Pennington, Socher & Manning, 2014).

**Figure 1:**
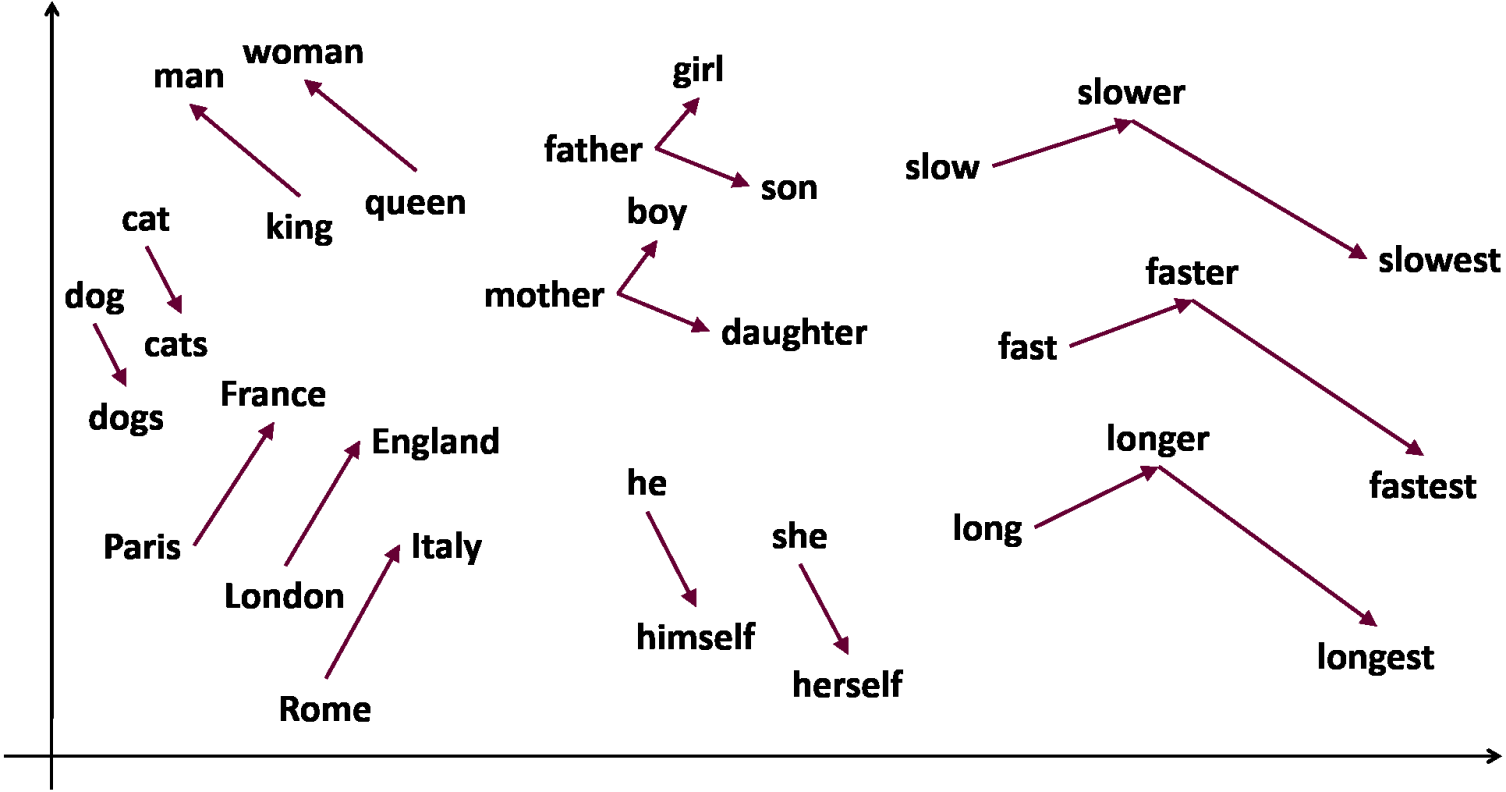
Word relationships through vector representation of words.

A conveniently word embedding trained model can accurately identify how likely it is that two words will occur simultaneously or, in other words, how likely it is that the two words can be used interchangeably. Figure 1 illustrates this concept of co-occurrence of words and the relationships that can be established between words. For example, “swimming” and “swam”, that are words semantically coherent, will have a high probability of co-occurrence, indicated by a low value of the cosine distance between their embeddings or vectors. Skip-gram (Mikolov et al., 2013) is one of the most commonly used word embeddings models, where analogous pairs of words tend to form parallelograms in the vector space. An example commonly used to illustrate this concept is the analogy: vec_“man”_ - vec_“king”_ + vec_“woman”_ ≈ vec_“queen”_.

Different distributional semantics models have been developed to generate embeddings, and these have proved to adequately capture the semantic properties of words, as long as sufficiently large corpora is used (Joulin et al., 2016; Mikolov et al., 2013). Several application scenarios have been tested including: classification of twitter streams (Khatua, Khatua & Cambria, 2019; Zhang & Luo, 2019), plagiarism detection (Tien et al., 2019), opinion mining on social networks (Nguyen & Le Nguyen, 2018; Rida-E-Fatima et al., 2019), recommendation systems (Chamberlain et al., 2020; Baek & Chung, 2021), mapping of scientific domain keywords (Hu et al., 2019), tracking emerging scientific keywords (Dridi et al., 2019), optimization of queries for Information Retrieval (Roy et al., 2019; Hofstätter et al., 2019) or sentiment analysis (Santosh Kumar, Yadav & Dhavale, 2021; Subba & Kumari, 2022). However, the great majority of such models has been developed for English corpora. It was only in recent years that the research community has also been focusing on other languages with rich morphology and different syntaxes (Hartmann et al., 2017; Rodrigues et al., 2016; Sun et al., 2016; Svoboda & Beliga, 2017; Svoboda & Brychcin, 2016; Turian, Ratinov & Bengio, 2010). Moreover, large datasets are required to enable achieving good performance. To reduce the need for large corpora, some authors have been proposing focusing on specific domains and having a corpus that correctly represents the use of specific words, showing that the specificity of the corpus has much more influence on word2vec results than its size (Dusserre & Padró, 2017).

## Research Objective and Contribution

This article describes a platform suitable for performing Portuguese keyword-based multimedia content searches by offering an online service that can determine the similarity of Portuguese terms. It incorporates a distributional semantic model trained with Word2Vec neural network and uses as input a Portuguese text corpus created from open-source newspapers. The model has been adequately parameterized to generate meaningful vector-space representations for each existing word in the input text corpus. Although this approach has already been adopted for other languages, especially English, very little attention has been dedicated to the Portuguese language. Additionally, very few works (Bruni, Tran & Baroni, 2011; Bruni, Tran & Baroni, 2014) have targeted the use of these models for multimedia content access or retrieval applications, as normally authors apply their solutions to textual content with the objective of detecting similarities between different excerpts of text. Whilst also applying our model to textual content, our work aims the identification of similar or related multimedia content and, thus, the text to analyse or to match are descriptions or tags that have been assigned to the multimedia content. The results that we have obtained indicate better performance on identifying related tags in comparison to solutions alike developed not only for Portuguese but as well as for English and other languages. Additionally, we also demonstrate that the size of the corpus can be significantly reduced, not impacting the performance of the model, without the need of creating domain-specific datasets.

Our main contributions are as follows:

• A new word2vec model for Portuguese that outperforms the existing state-of-the-art solutions for several languages, having as input a much smaller dataset (http://pt2vec.inesctec.pt/#modalInfo, http://pt2vec.inesctec.pt/files/model).
• A new dataset for the Portuguese language (https://doi.org/10.5281/zenodo.6396798).
• A publicly available API made available for the technical/scientific community and that enables using this model by exposing a REST interface.
• A web-based application to enable a user-friendly access to the model (http://pt2vec.inesctec.pt).
• A cloud-network-based visualization tool of word embeddings (http://pt2vec.inesctec.pt/projector/).

This article is structured in the following way: ‘Related Work’ starts by describing the most commonly used algorithms to train the desired distributional semantic models, then proceeding to present relevant work conducted by the research community using such algorithms. ‘Proposed Solution’ section describes the process used to create the Portuguese text corpus and the procedures implemented by the authors to train their model using the Word2Vec model and the Portuguese text corpus. It concludes by presenting the experiments and results obtained. ‘PT2VEC: A Portuguese Word2Vec Online Service’ provides an overview of the developed platform, describing the defined API and illustrating its usage. Finally, ‘Conclusions’ draws the concluding remarks for the article.

## Related Work

Deep learning methods for language processing owe much of their success to neural network language models. Words are represented as dense real-valued vectors and such representation is referred to as word embeddings given that they embed a vocabulary into a relatively low-dimensional linear space. One of the earliest ideas of distributed representations is presented in Hinton (1986) and has been applied to statistical language modeling with considerable success. These word embeddings have shown to improve performance in a variety of natural language processing tasks including automatic speech recognition, information retrieval, document classification, *etc*. The training is performed over a large corpus, typically in a totally unsupervised manner, using the co-occurrence statistics of words. Word vectors are typically obtained as a product of training neural network language models to predict the probability distribution over the next word. The learned word embeddings explicitly capture many linguistic regularities and patterns, such as semantic and syntactic attributes of words. Therefore, words that appear in similar contexts, or belong to a common topic (*e.g.*, country and city names, animals, *etc*.), tend to form a cluster. In Joulin et al. (2016), Mikolov et al. (2013) it has been demonstrated that word embeddings created by a recurrent neural network and a related model, the Word2vec, exhibit an additional linear structure that captures the relation between pairs of word. The use of simple vector arithmetic allows solving analogy queries such as “man is to king as woman is to?” In this example, “queen” happens to be the word whose vector V_queen_ is the closest approximation to the vector V_woman_ - V_man_ + V_king_ (Lee, 2015). Different algorithms and techniques have been developed in recent years to generate word embeddings from text data. All of them rely on the assumption that words that appear in similar context have similar meanings. The resulting models produce word vectors that can also be used to solve analogy queries. State-of-the-art developments have been focusing recently on other languages besides English.

### Word embeddings and topic modelling

#### Word2vec

**Figure 2:**
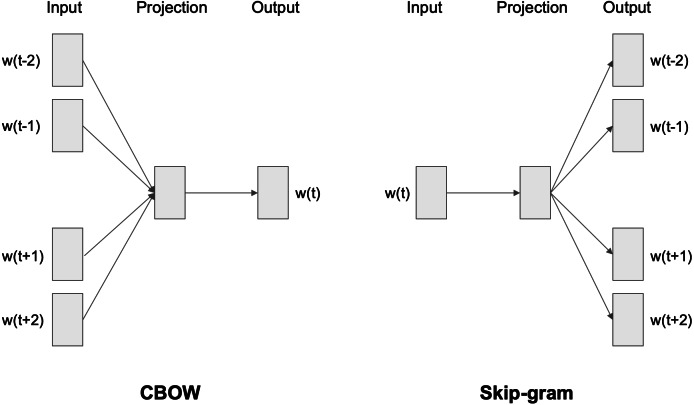
CBOW and skip-gram architectures.

Word to vector (Word2vec) is an efficient, intelligent algorithm for word embeddings generation (Joulin et al., 2016; Mikolov et al., 2013). It uses a neural network composed of three layers: input, hidden, and output. The main idea behind Word2vec is to take a large volume of text in one specific language and embed each vocabulary word as a vector in a vector space in such a way that the mathematical operation of vector addition has some connection to the meanings of the words. Word2vec computes vector representations of words using two different techniques: the continuous bag-of-words (CBOW) and the skip-gram architecture, represented in Fig. 2. In the CBOW approach, the model predicts the current word, from a window surrounding context words, by using both the n words before and after the target word w. In the skip-gram model, instead of using the surrounding words to predict the center word, it uses the center word to predict the surrounding words. According to Mikolov (Joulin et al., 2016; Mikolov et al., 2013), this architecture works well with a small amount of training data and performs a fair representation of rare words and phrases.

#### GloVe

GloVe (Global Vectors) (Pennington, Socher & Manning, 2014) is an unsupervised learning algorithm for obtaining vector representations for words. Training is performed on aggregated global word-word co-occurrence statistics from a corpus and the resulting representations showcase interesting linear substructures of the word vector space. Glove is essentially a count-based model, where a co-occurrence matrix of the entire training set is first built. Each entry in this co-occurrence corresponds to the simultaneous observation frequency of the target word (rows) and context word(columns). This matrix is factorized to yield a lower dimension (word x features), where each row now yields a vector representation for each word. The goal is to minimize the reconstruction loss and find the lower-dimensional representations to explain most of the variance in the high-dimensional data (Bhardwaj, Di & Wei, 2018). It considers the entire vocabularies’ bias terms and these learnable bias terms give an extra degree of freedom over Word2Vec and FastText (Liu, Ishi & Ishiguro, 2017).

#### FastText

FastText (Inc., 2020) is a library for efficient learning of word representations and sentence classification. Its main contribution is the idea of modular embeddings where, instead of computing an embedded vector per word, a vector is computed for subword components, usually n-grams; these are later combined by a simple composition function to compute the final word embeddings. This approach has the advantage of creating a smaller vocabulary making the algorithm computationally more efficient. Moreover, due to the subword information efficiency, the morphological variations keep most of their common components and have slight changes applied to their embeddings based on the differences, such as different prefix or suffix. In FastTex, as well as in Word2Vec, the training process works towards creating a model to predict words around the given input words.

### Applying the models to different languages

Many researches have investigated the behavior of the methods described above for the English language, but little attention has been dedicated to other languages. Only recently, some experiments have been described for other language datasets, including Portuguese (which is the 6th most spoken language in the world), and new mechanisms for validation have been proposed.

The work presented in Rodrigues et al. (2016) describes the creation and open distribution of word embeddings for Portuguese. Embeddings were evaluated and compared to the original English models based on the analogy dataset questions that were entirely translated by the authors to Portuguese. To enable comparing results, this analogy test set will also be used as our evaluation method. The authors have found that with their Portuguese model it was possible to achieve very similar results when compared to the state-of-art models for English. Results were evaluated using different parameter settings for the skip-gram model and reached adequate performance, with 52.8% accuracy in a restricted evaluation considering only the most frequent words and 37.7% accuracy for the entire vocabulary.

A large Portuguese corpus was gathered and described in Hartmann et al. (2017), including both Brazilian and European variants. It was used for training and evaluating different word embedding models (FastText, Glove, Wang2Vec and Word2Vec). The evaluation was performed intrinsically on syntactic and semantic analogies, and extrinsically on Part-of-speech (POS) tagging and sentence semantic similarity tasks. The intrinsic evaluation results on syntactic and semantic analogies for Word2Vec and skip-gram for European Portuguese have reached 33.5% accuracy.

Other languages have also been tackled by researchers. In Svoboda & Beliga (2017) the authors evaluated their Croatian model using Word2Vec and FastText. A new word analogy in Croatian was created based on the original English version with some modifications in the analogies’ categories. Additional word similarities were also created. The models were trained and compared using CBOW and skip-gram as word representation models with results presenting meaningful word representation with 32.03% and 33.89% accuracy, respectively, when considering the Word2Vec model. Three resources for evaluating the semantic quality of Finnish language distributional models were presented in Venekoski & Vankka (2017) by using semantic similarity, word analogies and word intrusion. Using a publicly available Finnish corpora, authors have translated all the resources for evaluation and compared the results with the original resources approach. Using the Word2Vec model with the skip-gram model, the authors have reached an accuracy of 36.55% on the analogy evaluation. Similar to the previous work, Svoboda & Brychcin (2016) explores the word embeddings methods in Czech. The authors have introduced a new dataset of word analogy questions that inspects syntactic, morphosyntactic and semantic properties of Czech words and phrases. Experiments show that Word2Vec CBOW model performs much better (32.5%) on word semantics than skip-gram (14.4%). Sun et al. (2016) describes the design of a document analogy task with new categories, for testing the semantic regularities in document representations. However, the presented approach only considers the semantic analogies, discarding the syntactic relations. Moreover, it creates new categories customized for the corpus content and uses the CBOW model instead of the skip-gram. This obviously affects the results making it not adequate for comparison purposes.

Table 1 provides a summary of the more relevant works described in the literature.

## Proposed Solution

### Creating a new portuguese training corpus

For the creation of the Portuguese dataset a web scraping was developed to extract news content from six renowned Portuguese online news websites. The resulting dataset includes titles, headlines and the article itself. A Python script using BeautifulSoup was our assistance to perform this web scraper along with some techniques that are discussed ahead. No lowercase/uppercase was performed enabling the distinction of specific cases such as Porto and porto (city of Porto and seaport in English). These words have different meanings, so they should provide different outputs on the building model.

**Figure 3:**
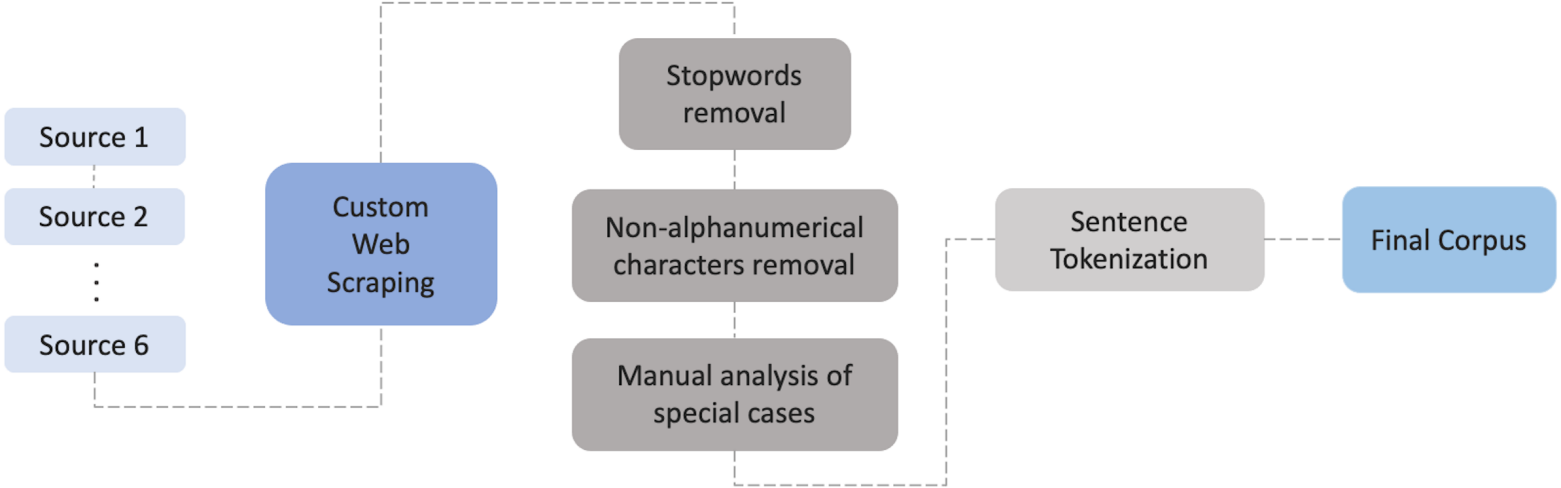
Workflow and steps for building the corpus.

The extracted text has been tokenized and cleaned by removing all non-alphanumeric characters and Portuguese stopwords. The stopwords file was augmented with words that are overly frequent in the dataset (mostly verbs and adverbs) but do not have an important definition/meaning. This was implemented by identifying the thousand most frequent words in the dataset. Also, a manual analysis of the dataset was performed in order to find words that were not formatted correctly, for instance due to wrong html formatting, and that appear together with other words, or numbers. Lastly, the processed data from the different sources were merged resulting in an initial corpus of 394,825,480 tokens. The full process is illustrated in Fig. 3.

**Table 1:** State-of-the-art review.

**WORD EMBEDDING MODELS**		
**Author**	**Model**	**Description**
(Hinton et al., 1986)	Multilayer Neural Network trained using Backpropagation	Original work on the use of neural networks to make explicit the semantic features of concepts and relations present in the data.
(Mikolov et al., 2013)	Word2Vec	Three layers neural network for obtaining vector representations of words.
(Pennington, Socher & Manning, 2014)	Glove	Unsupervised learning algorithm for obtaining vector representations for words.
(Inc., 2020; Bojanowski et al., 2017)	FastText	Extension of the continuous Word2Vec skip-gram model, taking into account subword information.
**WORD EMBEDDING APPLICATIONS**		
**Author**	**Application**	**Approach/Contribution**
(Subba & Kumari, 2021)	Sentiment Analysis	Combination of Word2Vec, GloVe and BERT.
(Kumar, Yadav & Dhavale, 2021)	Sentiment Analysis	Accuracy comparison of different pre-trained and untrained word embedding models.
(Hofstätter et al., 2019)	Information Retrieval	Adaptation of the skip-gram model’s vectors using global retrofitting.
(Dridi et al., 2019)	Tracking emerging scientific keywords	Temporal Word2Vec to track the dynamics of similarities between pairs of keywords.
(Hu et al., 2019)	Mapping of scientific domain keywords	Word2Vec to enhance the keywords with semantic information.
(Chamberlain et al., 2020)	Recommendation systems	Word2Vec hyperparameters optimization.
(Baek & Chung, 2021)	Recommendation systems	Word2Vec for social relationship mining.
(Roy et al., 2019)	Information Retrieval	Optimization of queries.
(Tien et al., 2019)	Plagiarism Detection	Multiple pre-trained word embeddings and multi-level comparison for measuring semantic textual relation.
(Zhang & Luo, 2019)	Hate Speech	Deep neural network structures serving as feature extractors for capturing the semantics of hate speech.
(Khatua, Khatua & Cambria, 2019)	Analysis of twitter streams	Contextual Word2Vec for classifying twitter streams
(Nguyen & Le Nguyen, 2018)	Opinion Mining on Social Networks	Convolutional N-gram BiL-STM word embedding model for sentiment analysis by capturing semantic and contextual information.
(Rida-E-Fatima et al., 2019)	Opinion Mining on Social Networks	Refined word embeddings model exploiting the dependency structures without using syntactic parsers.
**MULTIMEDIA DOMAIN**		
**Author**	**Modalities**	**Contribution**
(Bruni, Tran & Baroni, 2011; Bruni, Tran & Baroni, 2014)	Text and Images	Multimodal semantic model combining text and image-based features.
(Pinto & Viana, 2013)	Text and Video	Use of semantic dictionaries to extract semantically related concepts in video metadata.
**APPLYING WORD EMBEDDING TO DIFFERENT LANGUAGES**		
**Author**	**Language**	**Model**
(Sun et al., 2016)	English	Word2Vec (CBOW)
(Joulin et al., 2016)	English	FastText
(Rodrigues et al., 2016)	Portuguese	Word2Vec (skip-gram)
(Hartmann et al., 2017)	Portuguese	FastText, GloVe, Wang2Vec, Word2Vecx (skip-gram)
(Svoboda & Beliga, 2017)	Croatian	Word2Vec (CBOW, skip-gram), FastText
(Venekoski & Vankka, 2017)	Finish	Word2Vec (skip-gram)
(Svoboda & Brychcin, 2016; Svoboda & Beliga, 2017)	Czech	Word2Vec (CBOW, skip-gram)

Gensim (Rehurek, 2019; Rehurek & Sojka, 2010), a Python library for topic modelling, document indexing and similarity retrieval with a large corpora will be used in a later phase for training and evaluation as it can perform natural language processing (NLP) and unsupervised learning on textual data, offering a wide range of algorithms: TF-IDF, random projections, latent Dirichlet allocation, latent semantic analysis, word2vec and document2vec. A significant advantage of gensim is that it enables handling large text files without having to load the entire file in memory.

Since Gensim’s word2vec (Rehurek & Sojka, 2010) expects a sequence of sentences as its input, a sentence tokenization has been performed directly on the dataset as a memory efficient approach. This allows us to improve the script processing time in order to train the model. The final tokenized dataset has generated 33,089,734 sentences.

### Training the model

**Figure 4:**
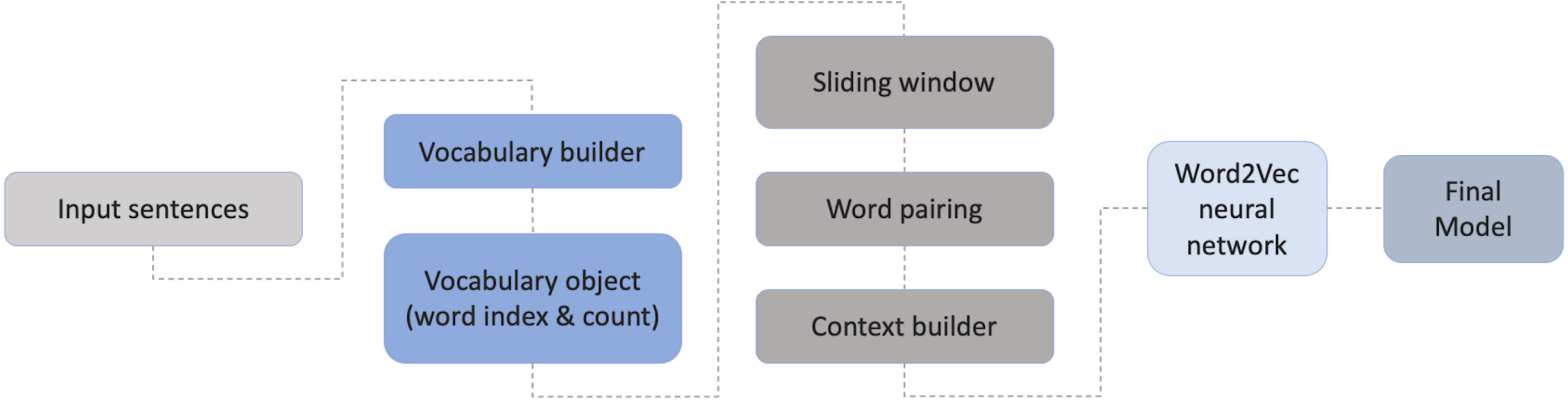
Steps for building the final model.

Gensim (Rehurek, 2019; Rehurek & Sojka, 2010), was used for training and evaluation, as it is flexible and intuitive to use. For the creation of the Portuguese word embeddings the skip-gram model was chosen in order to be able to compare the results with the original English evaluation (Joulin et al., 2016; Mikolov et al., 2013) and other language-based experiences, including Portuguese (Hartmann et al., 2017; Rodrigues et al., 2016; Sun et al., 2016; Svoboda & Beliga, 2017; Svoboda & Brychcin, 2016; Venekoski & Vankka, 2017). In this technique, and given a set of sentences, the model loops through the words of each sentence and tries to predict its neighbors (*i.e.,* its context), within a certain range before and after that word (the window size). Word2Vec model starts by building a vocabulary by extracting the unique words on the dataset and creating an object that contains the word index and its count. The next level will be responsible to build the context by converting the words into vectors. By taking all the words in a pre-defined window size, this will create word pairings that will feed the neural network. This will lead to an increase of context of the center words and the pairs, thus helping to identify the relevant meaning of the word. The final Word2Vec uses a two-layer neural network. The full process is illustrated in Fig. 4.

The vectorized words do not contribute to find similarities, since the distance between each word is the same. As so, the architecture of Word2Vec allows to create weights for the input words. By using backpropagation, the weights are updated for each combination of words based on the context of each phrase. At the end, on the output layer, the Softmax function creates the probability distribution. Additional modifications and techniques that affect both training speed and quality can be used by Gensim. More detailed information can be found at (https://radimrehurek.com/gensim/models/word2vec.html).

Multiple trials for hyperparameters tuning were performed, resulting in the final model that enables achieving the best results.

### Word analogy evaluation

Mikolov (Joulin et al., 2016; Mikolov et al., 2013) suggested capturing the relations between words as the offset of their vector embeddings. For the evaluation of the semantic reliability of our model, analogies have been used: The Google Analogy Dataset contains 19,544 questions in the form a is to b as c is to d and it is divided into semantic and syntactic sections. Based on the analogy type, each section is further divided into subcategories. In the test task, a well-performing model is expected to estimate the correct word d given vectors of words a, b and c, obtained from the linear operation Wb + Wc - Wa, by estimating the most similar word vector to that operation. The evaluation model expects all the three input vectors to exist in the vocabulary. If one of then does not appear in this vocabulary, the evaluation model assumes that an erroneous decision for the predicted Wd vector was taken. This will have a negative impact on the final accuracy measure that could be improved if those cases were identified and eliminated from the test set. The capacity of the model to capture known semantic relations is measured by the overall percentage of correct analogies (Venekoski & Vankka, 2017).

Although the original analogy dataset is very helpful to evaluate an English model, it is not directly suitable for other languages. To overcome this problem, we have used the only publicly available word analogies for Portuguese (NLX-group, 2020). It was translated from the original English dataset to Portuguese by native Portuguese-speaking language experts. In this process, it was taken into consideration that some English words could not be accurately translated into a unique Portuguese word and it was not supported by the original evaluation vector composition. This resulted in a Portuguese analogy dataset of 17,558 analogies which were used to calculate the accuracy of our word embedding model. The performance of our model was compared against the original Word2Vec implementation for English (Joulin et al., 2016; Mikolov et al., 2013) and the previous published work for Portuguese (Rodrigues et al., 2016). The evaluation was performed using two approaches to keep an equivalent methodology: (1) vocabulary restricted evaluation, which ignores all questions containing a word not found in the top 30.000 words; (2) unrestricted evaluation, considering all the words.

The model was trained under similar conditions to those previously published in the literature (Joulin et al., 2016; Rodrigues et al., 2016) so that results could be fairly compared (obviously, considering a bigger window size or increasing the number of epochs could enable better results but at the expense of computational costs).

Following also the approach used by previous work, the final model used is the one that enables achieving the best results for our dataset: window distance of 5; a vector size of 300; an initial learning rate of 0.025; a threshold of 1e−5; a negative sampling of 15; and a total word frequency lower than 200 (minimum count). For these parameters, a vocabulary of 72,757 unique words was considered.

Table 2 presents the results obtained by our model. The low performance for some of the categories in the semantic section, as currency and city-in-state, can easily be explained by the fact that this data does not exist in the created dataset: it is highly unlikely that the collected Portuguese local news include references to states in United States of America as well as worldwide currency. The worst results in the syntactic section are for the gram3-comparative and gram4-superlative categories. This fact is directly associated with the pre-processing done on the text collected from the news: the words in these categories have been treated as stopwords and removed from the corpus making the model failing if exposed to them. The idea behind this decision was that words like mau, grande, pior, forte, fácil, *etc*. (bad, big, worst, strong, easy in English) are not relevant for the purpose of describing and tagging content and could then be removed. However, this decision has a negative impact on the evaluation process that includes this kind of concept.

**Table 2:** Accuracy without and with restriction.

Type	Accuracy without restriction	Accuracy with restriction
capital-common-countries	90.5% (380/420)	90.1% (308/342)
capital-world	82.8% (1775/2143)	87.9% (1012/1151)
currency	7.3% (13/178)	8.5% (9/106)
city-in-state	31.0% (239/772)	31.7% (60/189)
family	50.3% (154/306)	58.3% (140/240)
gram1-adjective-to-adverb	18.7% (103/552)	25.8% (62/240)
gram2-opposite	35.7% (65/182)	51.4% (37/72)
gram3-comparative	0% (0/0)	0% (0/0)
gram4-superlative	19.4% (14/72)	0.0% (0/2)
gram5-present-participle	51.7% (310/600)	61.4% (129/210)
gram6-nationality-adjective	92.8% (1140/1229)	96.0% (930/969)
gram7-past-tense	51.1% (608/1190)	49.3% (272/552)
gram8-plural	45.4% (509/1122)	51.5% (511/992)
gram9-plural-verbs	63.6% (267/420)	65.0% (273/420)
TOTAL	60.7% (5577/9186)	68.2% (3743/5485)

**Figure 5:**
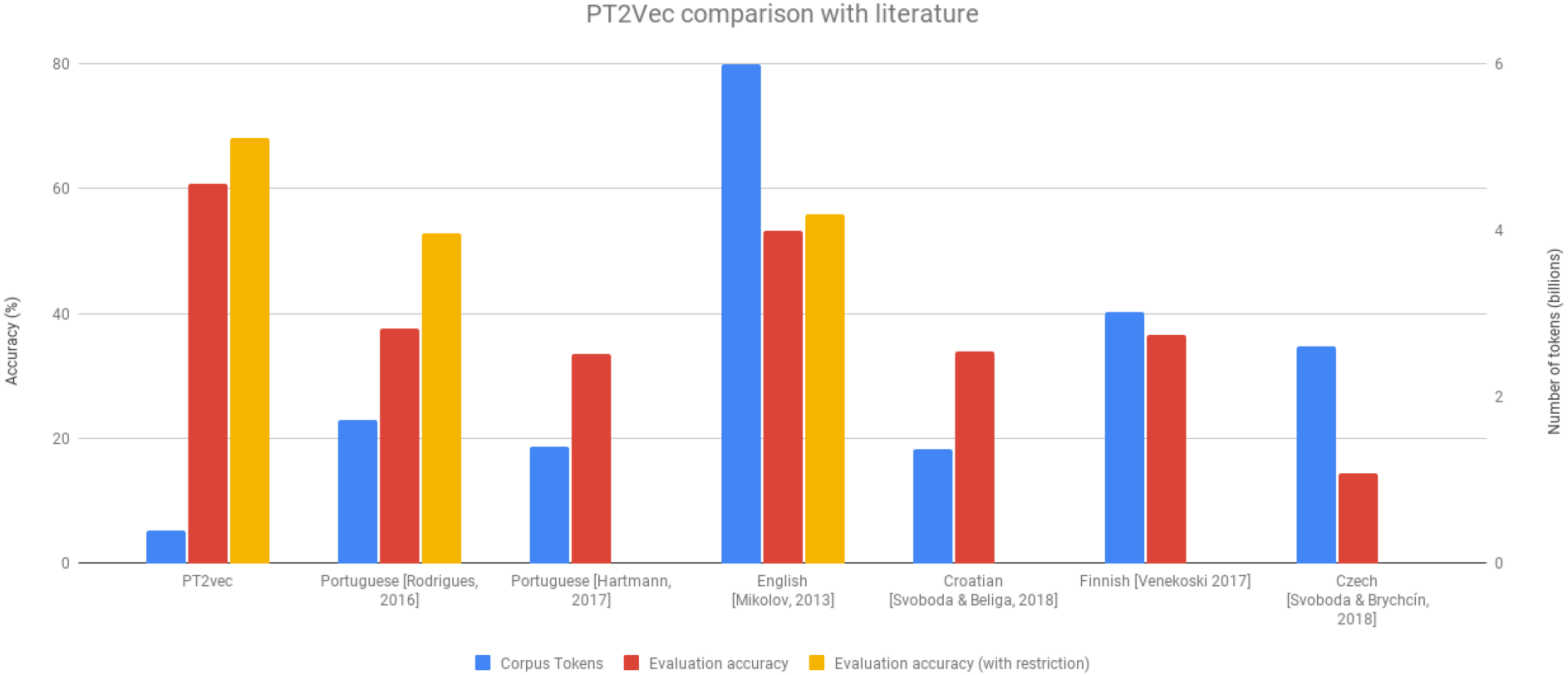
PT2Vec *vs* SoA approaches.

Figure 5 enables comparing our solution with other SoA approaches. Although our dataset is significantly smaller than any of the others (at least five times less tokens), it outperforms all the others on the accuracy achieved. This is also true when considering the methodology previously used in one of the works of excluding the less frequent vocabulary. This dataset reduction is also quite relevant as it avoids the need of collecting huge amounts of data and enables decreasing the computational costs.

## PT2VEC: A Portuguese Word2Vec Online Service

The good results achieved by our model make it relevant for being used by external systems for similarity specific tasks, such as find the most similar words, find the similarity between two words, *etc*. In order to make its functionalities available and open to the community, an online service was implemented using CherryPy for a fast and minimalist Python web framework. For the model load and interaction, Gensim was used. Additionally, a cloud-network-based visualization tool of word embeddings is also provided in order to enable navigating and searching for words similarity in a three-dimensional space.

Besides being very fast, our platform explores all the methods available in the Gensim framework (Rehurek, 2019), enabling the creation of four different queries: get the most similar words; find which word does not fit in a group of related words; compute the cosine similarity between two words; compute word pairs.

### Publicly available API

A REST API was implemented for method invocation, with the results being returned in a JSON formatted output. This allows users to link the API services with their own applications. Multiple methods have been implemented to interact with the server, as described in Table 3. Examples below show the use of the most_similar method to compute the word analogy pair having as positive words king (rei) and woman (mulher), and as negative word man (homem). The request can be performed using an HTTP GET method or using a cURL query.

**Table 3:** Portuguese word embeddings PT2VEC API.

Method	HTTP request	Description	Parameters
most_similar	GET	Find the top-N most similar entities to the positive list of words	positive[] (required) topn (required)
		Find the element that enables creating the analogy par	positive[] (required) negative[] (required) topn (required)
most_dissimilar	GET	Detect from a list, the word that does not go with the others	words (required)
similarity	GET	Compute cosine similarity between two entities, specified by their string id	word1 (required) word2 (required)

**Figure 6:**
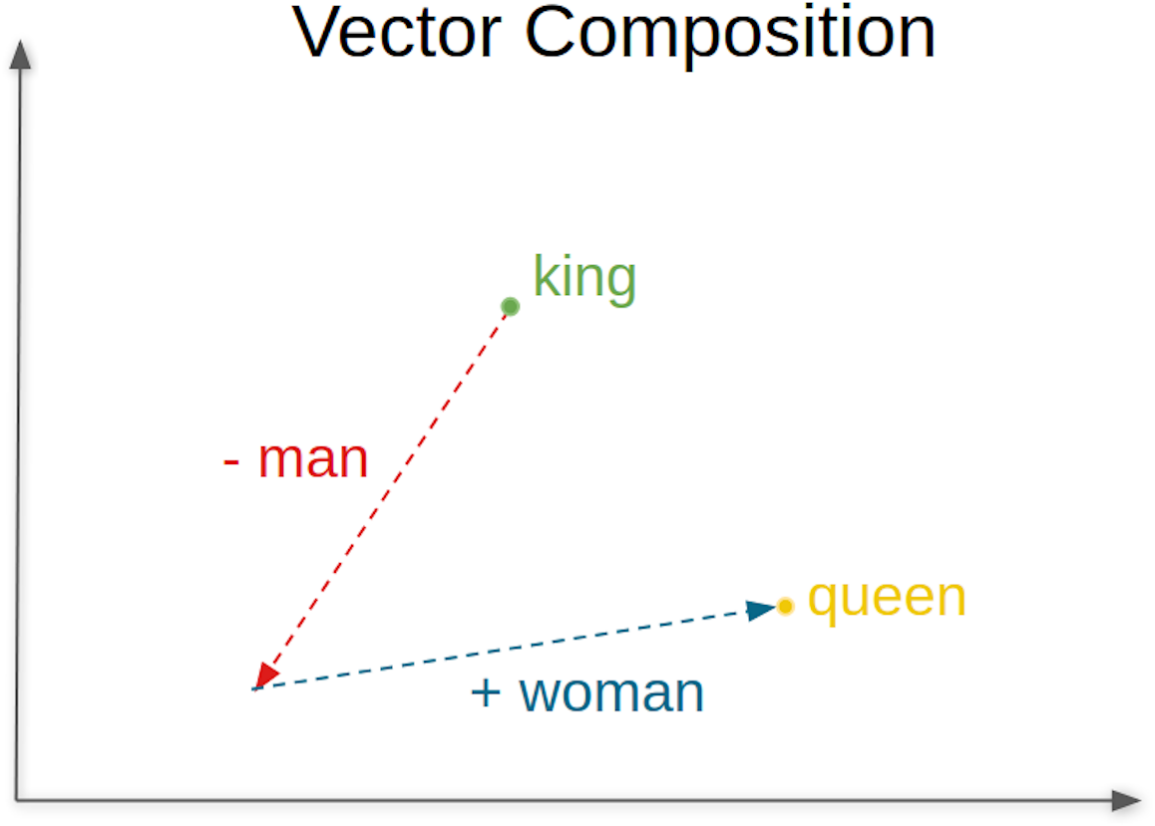
Vector composition for the analogy king/man queen/woman.

The answer includes the properties time and similars. The former indicates the time in milliseconds to process the request, and the later is a vector containing the computed list of words displayed in descending order of similarity accuracy (rainha =queen; monarca =monarch; reis =kings; princesa =princess). For the example presented, the proposed top word is rainha. Figure 6 represents the vector composition for this words’ analogy.

### Web-based application

**Figure 7:**
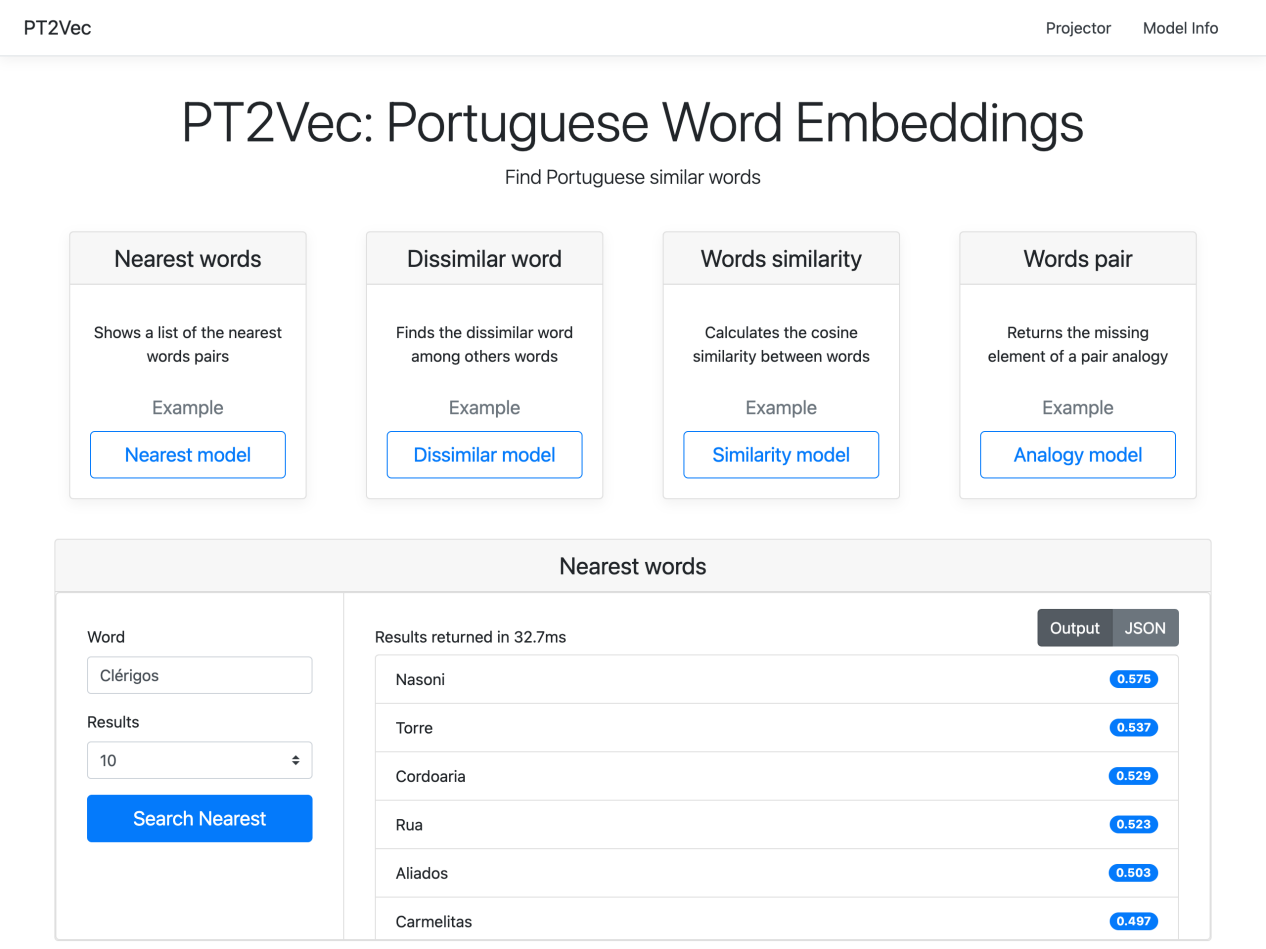
PT2Vec web interface.

PT2Vec is also available through an interactive web application (http://pt2vec.inesctec.pt), as illustrated in Fig. 7. All the four services can be used and enable introducing the required parameters. In the example presented, the output of a query to the Nearest Words service is shown. The chosen model applies the most_similar method with a list of words that contribute positively as parameter. The service allows users to display a predefined number of results that can be toggled between the representation as in the image, or by a JSON structure.

 To guarantee that the word belongs to the corpus used to create the PT2Vec model, an auto-complete functionality for suggestion was implemented. Taking into account that this model was trained as case sensitive, the query words and results are also case sensitive.

### Embedding projector

A cloud-network-based visualization tool of word embeddings is also included in the PT2vec platform. It is based on Tensorflow, an open-source library for numerical computation using data-flow graphs. Tensorflow includes a suite of visualization tools called TensorBoard that can be used to visualize a TensorFlow graph, plot quantitative metrics about the execution of a graph, and show additional data. This library is usually used as a tool to graphically visualize the progress of the model.

This framework was adapted to enable projecting embeddings from a word2vec model to a lower dimensional space. The mesmerizing feature of TensorBoard was used to implement this functionality of projecting a word cloud by taking the high dimensional vectors and project them into a lower dimensional space. The dataset dimensionality can be reduced by using PCA, t-SNE or a custom dimensionality reduction technique.

**Figure 8:**
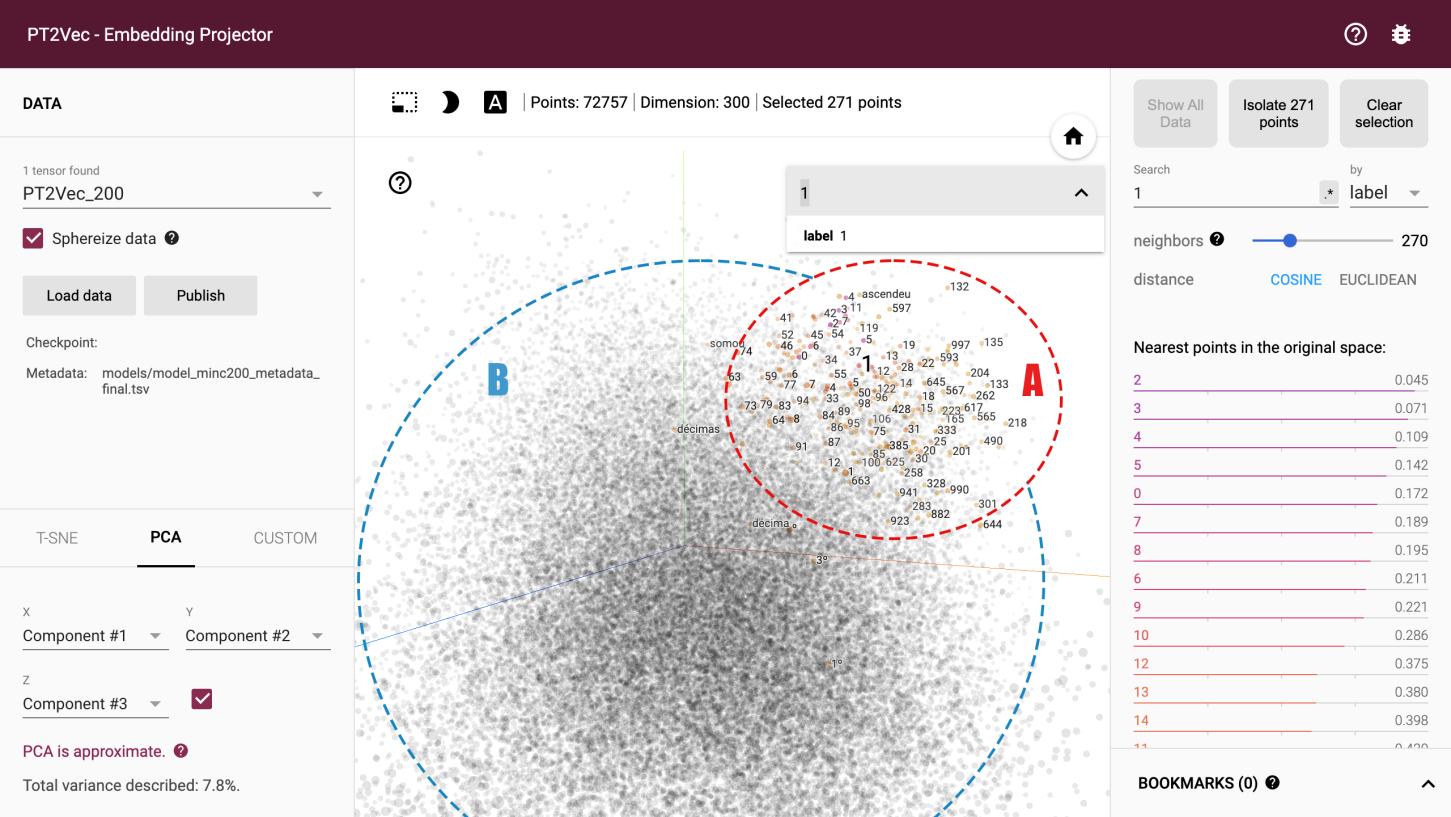
Full PT2Vec visualization.

For the creation of the embedding projector, word-vectors from the Word2Vec model were converted to the Tensorflow TSV format using the Gensim script word2vec2tensor. This enabled the creation of a Tensorflow 2D tensor containing the word-vectors and metadata format containing words. Figure 8 shows the full graph visualization of PT2Vec with 72,757 different words represented as points. Embeddings can be visually explored by zooming, rotating, searching and panning, using natural click-and-drag interactions. Hovering the mouse over a point will show any metadata for that point as shown in Fig. 8. Nearest-neighbor subsets can also be inspected. Clicking on a point causes the right panel to list the nearest neighbors, along with distances to the current point.

The embedding projector enables rapidly identifying different regions in the dataset. The largest cluster in the example (cluster A) includes mainly words while the smaller (cluster B), on the right, represents mostly numbers, while still keeping also words that are somehow related with them. A closer look on this cluster shows that words like “euros”, “quilo”, “totais”, “milhões” (in English “euros”, “kilo”, “totals”, “millions”), *etc*. are included and present an high degree of correlation with numerical values. This illustrates an additional feature of this representation given that extra information can also be inferred, enabling identifying words that relate to numerical values. The full model includes several other distinct clusters that enable finding additional relations in the projection.

**Figure 9:**
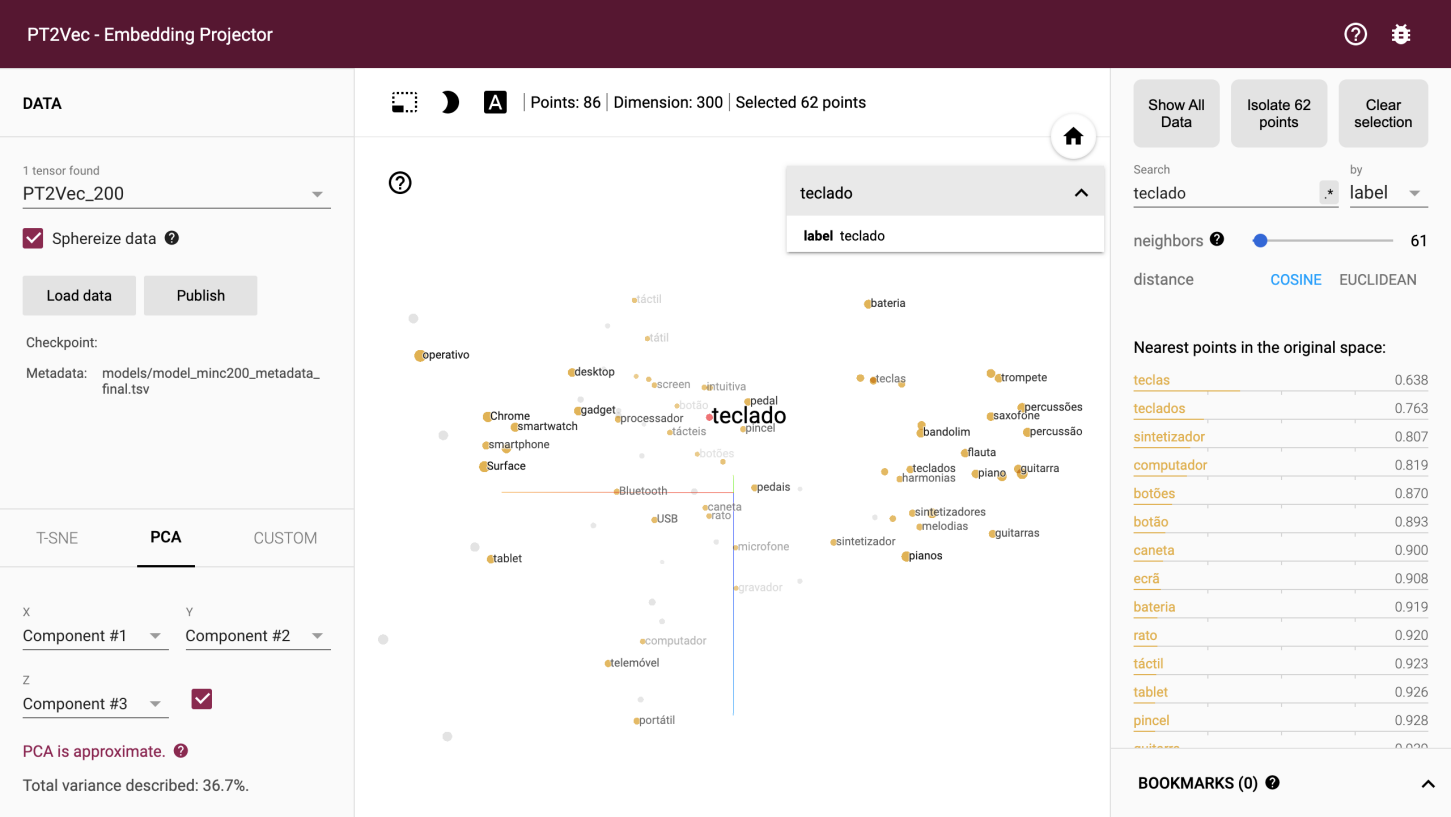
PT2Vec navigation tool.

Due to the size of the embedding model, restricting the view to a subset of points and performing projections only on those points enables a more focus view of the relations (Fig. 9). The tool enables a non-linear navigation in the model either by clicking in one of the points in the center panel or by selecting one of the words in the right-side listing.

**Figure 10:**
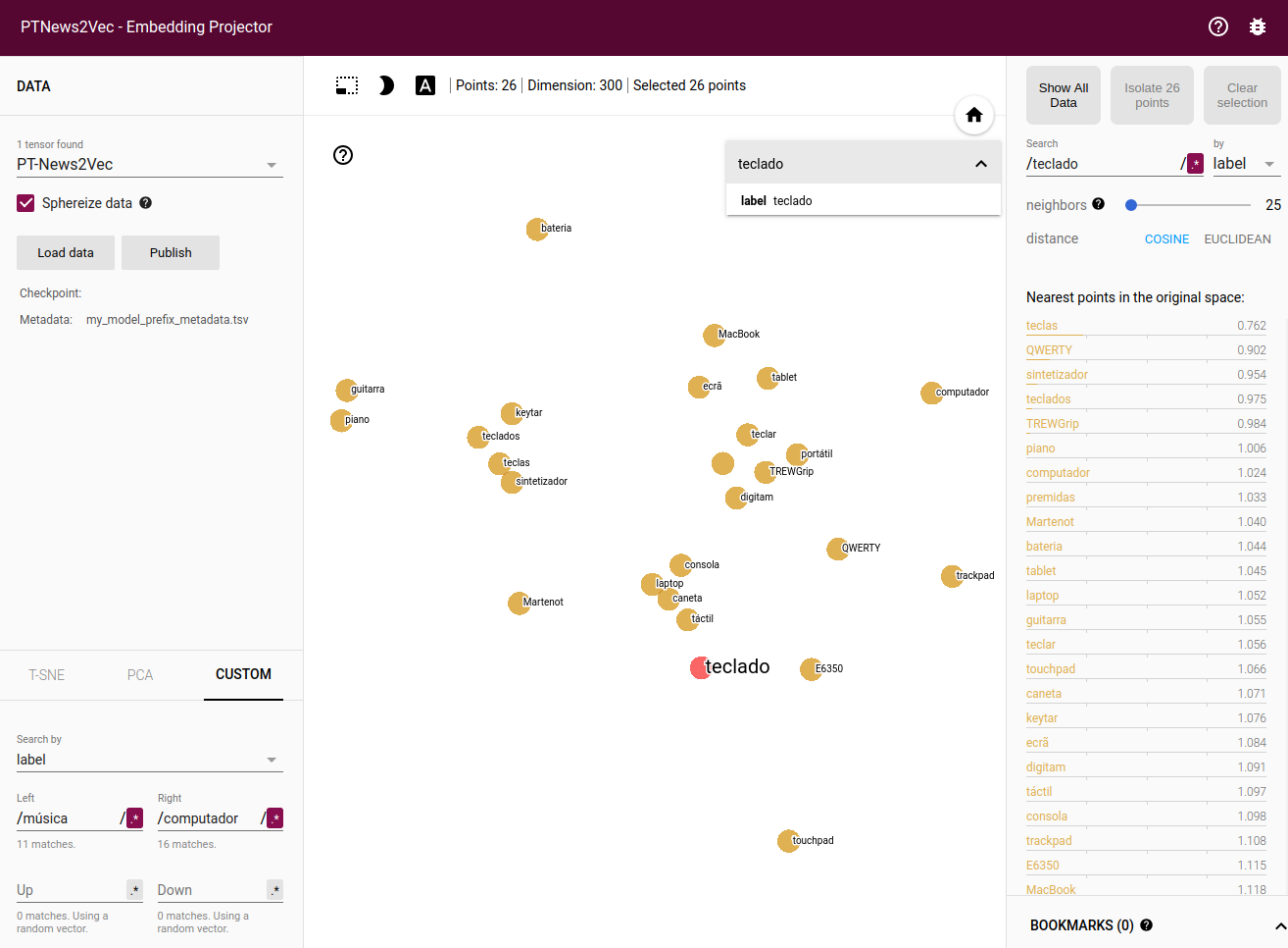
Word contextualization in different domains.

Custom projection controls offer a powerful linear projection onto a horizontal and a vertical axis enabling the customization of the information to be presented by specifying the labels. The projector tries to find all the points whose label matches the assigned keywords and computes the centroid which is used to define the axis and a random vector for the y axis. Figure 10 shows a filter using custom projection for the nearest neighbors of the word teclado (keyboard in English) projected onto the música and computador concepts (music and computer in English) as an x axis. As a result, one finds on the right side of the screen “processador” (microprocessor), “smartphone”, “gadget”, *etc*. (concepts related to the computer world), while, on the left, words related to the music field, as guitar and piano are displayed. This functionality enables disambiguating words that have different meanings depending on the context.

## Conclusions

This article presents a word embedding model for the Portuguese language, the sixth most spoken language in the world. The model was built using the Word2Vec skip-gram algorithm to extract word embeddings from a corpus of online Portuguese newspapers.

The performance of the developed model was evaluated using a Portuguese analogies dataset (NLX-group, 2020), revealing that it outperforms existing state-of-the-art models, achieving accuracies of 60.7% and 68.2% for non-restricted and restricted vocabulary, respectively. This is an important improvement when compared to SoA works as they all show results below 40%. Our better results can be explained by two factors: the approach used to create the dataset that guarantees a high standard of quality of the data used for training and the careful fine tuning of the model’s hyper-parameters.

Ours results are extremely auspicious, given that they were obtained using a rather limited-size dataset, especially when compared with the size of the datasets used by similar works—at least five times smaller than those ones. Accordingly, it is plausible to expect that even better results are likely to be obtained simply by increasing both the size of the dataset as well as its diversity, with online newspapers. In the future we intend to analyse the impact of increasing the dataset both in what concerns the accuracy achieved as well as the additional computational costs introduced.

Our model is made available as an open service to the community as a web page and an API. The web interface exposes four different methods and the API allows developers to integrate it on their applications freely. A cloud-network-based visualization tool of word embeddings is also available allowing an intuitive user interaction with the model.

## Supplemental Information

10.7717/peerj-cs.964/supp-1 Supplemental Information 1 PT2Vec Software Code Software code that implements the different functionalities described in the article.

## Additional Information and Declarations

### Data Availability

The following information was supplied regarding data availability: API and online service to access the publicly available Pt2Vec model is available at: http://pt2vec.inesctec.pt/
Information on the W2V Model parameters is available at PT2Vec: Portuguese Word Embeddings:
http://pt2vec.inesctec.pt/#modalInfo
The dataset is available at Zenodo: Paula Viana, & José Pedro Pinto. (2022). PT2vec - A Portuguese text corpus created from online newspapers [Data set]. Zenodo. https://doi.org/10.5281/zenodo.6396798.
