# Supplementary material for: Improving word embeddings in Portuguese: increasing accuracy while reducing the size of the corpus
Source: PeerJ Comput Sci. 2022 Jul 18;8:e964. doi: 10.7717/peerj-cs.964 (PMC9301597; doi:10.7717/peerj-cs.964)
Supplement: Supplemental Information 1 — Software code that implements the different functionalities described in the article. [file peerj-cs-08-964-s001.zip › pt2vec-code/index.html]

PT2Vec - Find Portuguese related words


##### PT2Vec

Model Info

# PT2Vec: Portuguese Word Embeddings

Find Portuguese similar words

#### Nearest words

- Shows a list of the nearest words pairs

#### Example

Nearest model

#### Dissimilar word

- Finds the dissimilar word among others words

#### Example

Dissimilar model

#### Words similarity

- Calculates the cosine similarity between words

#### Example

Similarity model

#### Words pair

- Returns the missing element of a pair analogy

#### Example

Analogy model

#### Nearest words

Word


A word is required.

Results

5
10
20
50

Please select a valid number.

Search Nearest

Output

 JSON

#### Dissimilar word

Words


At least 3 words are required.

Search Dissimilar

Output

 JSON

#### Words similarity

Words


Two words are required.

Search Similarity

Output

 JSON

#### Words Analogy

Words Analogy


Three words are required.

Results

1
2
3

Please select a valid number.

Search Analogy

Output

 JSON

##### W2V Model Information

×

This demo was trained with Word2Vec and Gensim using data from Portuguese news websites.

Model was build with a total 248,537,667 tokens, from 20,683,339 sentences and a vocabulary of 345,107 unique words with at least 5 occurrences.

Parameteres used to train:

- Training model: Skip-Gram
- Negative sampling: 15
- Number of training threads: 4
- Number of training iterations: 10
- Min word frequency: 5
- Vector size: 300
- Max skip length: 5
- Threshold for occurrence of words: 1e-05
- Starting learning rate: 0.025

Close

© INESC TEC
