# Supplementary figures and images for: Improving word embeddings in Portuguese: increasing accuracy while reducing the size of the corpus
Source: PeerJ Comput Sci. 2022 Jul 18;8:e964. doi: 10.7717/peerj-cs.964 (PMC9301597; doi:10.7717/peerj-cs.964)

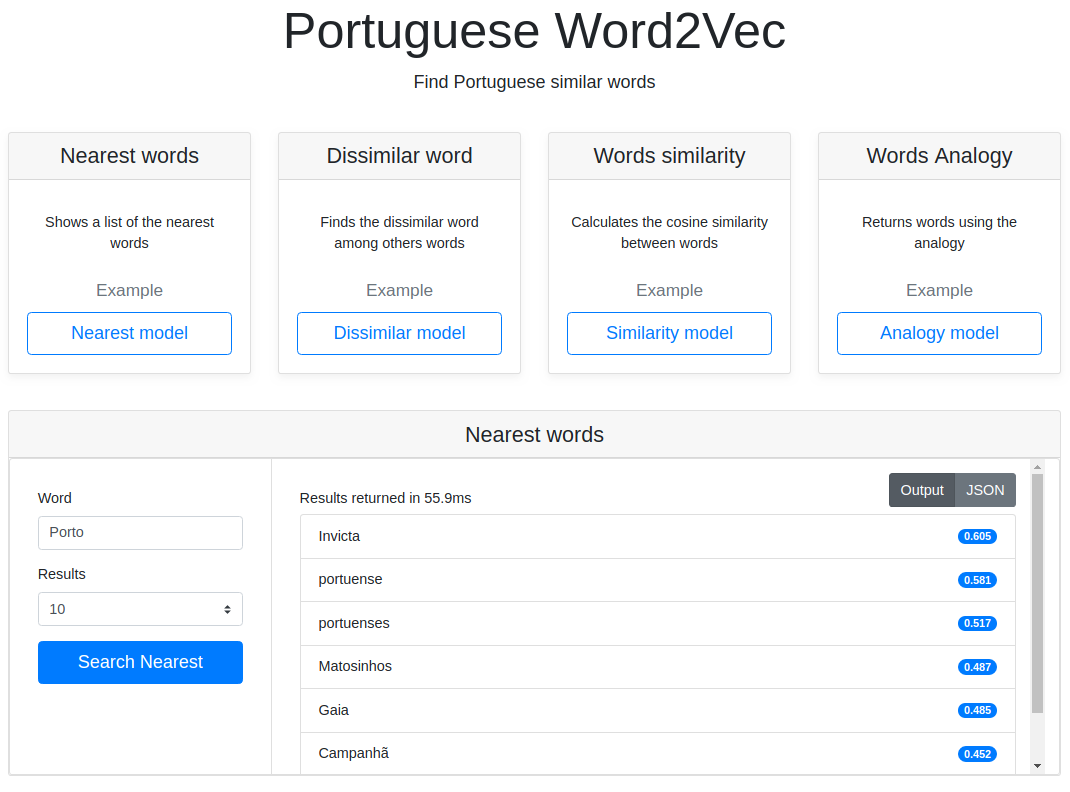

Supplement: Supplemental Information 1 — Software code that implements the different functionalities described in the article. [file peerj-cs-08-964-s001.zip › pt2vec-code/images/PT2Vec.png]
